# Reflectance, absorbance and transmittance spectra of bermudagrass and manilagrass turfgrass canopies

**DOI:** 10.1371/journal.pone.0188080

**Published:** 2017-11-27

**Authors:** Marco Volterrani, Alberto Minelli, Monica Gaetani, Nicola Grossi, Simone Magni, Lisa Caturegli

**Affiliations:** 1 Department of Agriculture, Food and Environment, University of Pisa, Pisa, Italy; 2 Department of Agricultural Sciences, University of Bologna, Bologna, Italy; United States Department of Agriculture, UNITED STATES

## Abstract

Leaves act as a primary organ for the interception of solar radiation and their spatial arrangement determines how the plant canopy interacts with light. Many studies have been carried out on the penetration of radiation into crops however to date, few results are available on turfgrasses, mainly due to the difficulties of introducing sensors into the turf without disturbing the natural position of the leaves. In the present research two warm season turfgrasses, hybrid bermudagrass (*Cynodon dactylon* × *transvaalensis)* 'Patriot' and manilagrass (*Zoysia matrella)* 'Zeon', were studied. The aim was to describe their canopy architecture grown with minimal disturbance to the natural arrangement of the leaves and stems, and to determine the potential effects of canopy architecture on light penetration and reflectance. Radiometric measurements were carried out at eight different profile levels of turfgrasses that were up to 12 cm tall. A LI-COR 1800 spectroradiometer with an optical fiber cable and a 7 mm diameter sensor was used. Measurements were carried out in the 390–1100 nm region at 5 nm intervals. The LAI value was higher for the manilagrass (9.0) than for the hybrid bermudagrass (5.6). The transmitted radiation was found to be closely dependent on downward cumulative LAI. Despite a more upright habit (mean insertion angle of 22.4° ±3.4), *Zoysia matrella* showed a higher NIR reflectance compared to Cdxt, which has a horizontal leaf arrangement (mean insertion angle 62.1° ± 9.6). The species studied showed substantial differences both in terms of phytometric characteristics and in the capacity to attenuate solar radiation.

## Introduction

Plants depend on solar radiation for the energy necessary to carry on photosynthesis and photomorphogenesis. For the interception of incoming radiation leaves act as primary organ and a number of properties have been reported to affect the leaf-light interaction such as cell structure [1], pigment content [2], and surface characteristics [3]. Light reaching every point within a canopy derives from two components: unfiltered radiation (direct or diffuse) and filtered radiation which is modified and attenuated as it passes through the vegetation. Incident radiation is only partially reflected by the external surface of a plant, while at the canopy level, light interacts extensively with a plant community with a multitude of reflection, refraction and diffusion phenomena which occur both within single leaves and the whole canopy. Incident radiation is therefore partly absorbed, transmitted and subsequently redirected outside the canopy as reflected light [4]. The radiation reflected by plant canopies brings a specific “spectral signature” and the analysis of reflectance spectra provides valuable information on the species [5] color [6; 7; 8] Leaf Area Index (LAI) [9; 10] chlorophyll content [11], drought stress [12; 13] and nutritional status [14; 15] of many crops and turfgrasses. Based on the interpretation of spectral reflectance instruments have been developed for the early detection of stress in plants or for large scale data acquisition [16; 17; 18].

The canopy architecture, however, may lead to substantial variations in the plants ability to interact with the incident radiation [19]. Canopy architecture is broadly defined as the amount and spatial arrangement of the aboveground plant organs [20; 21]. Parameters such as the leaf area index (LAI) [22; 23; 24], shoot density [20], specific leaf weight (SLW) [25], have been used in previous studies to describe the amount of plant tissue over a given soil surface. On the other hand, the spatial arrangement has often been described by the determination of leaf angles, sometimes referred to as leaf orientation or leaf inclination [26]. Leaf biochemistry, namely pigments, and canopy architecture, have been shown to be distinct sources of variability in reflectance spectra. Pigment content and composition affect reflectance in the photosynthetically active radiation (PAR) range (400–700 nm), whereas reflectance in the near infrared (NIR) range (750–1100 nm) is governed mainly by canopy architecture, cell structure and leaf inclination [27].

A large number of studies on the penetration of radiation into crop canopies have focused on field crops [28; 29; 30; 31; 32; 33; 34; 35; 36]. Given the difficulty of using radiometric equipment in turf conditions, only limited research work has been carried out on turfgrass [37; 24;4] with most radiometric studies being based on reflectance measurements [38].

Little is known about the light penetration within turf canopies, and the study of both the reflectance and transmittance of light in turfgrass species may provide insight into the growth and adaptive characteristics [39].

The aim of the research was to provide more extensive knowledge of light penetration into turfgrasses and to determine the potential effects of canopy architecture on light-plant interactions.

## Materials and methods

The trial was carried out from March to August 2015 in S. Piero a Grado, Pisa, at the Department of Agriculture, Food and Environment (DAFE) of the University of Pisa (43°40’ N, 10° 19’ E, 6 m. a.s.l.), on mature stands of *Zoysia matrella* (L.) Merr. cv 'Zeon' (Zm) and *Cynodon dactylon x transvaalensis* Burtt.-Davy ‘Patriot’ (Cdxt).

The swards were established on a calcaric fluvisol (coarse–silty, mixed, thermic, Typic Xerofluvents) with pH 7.7 and 22 g kg^−1^ of organic matter.

In February 2015, winter brown leaves were removed by scalping at 1.0 cm. In 2015, no fertilizer was applied to the turfs before the trial started. Mean air temperature and rainfall during the trial period are reported in Table 1.

**Table 1:** Average monthly minimum and maximum air temperature and monthly cumulated rainfall from March to August 2015.

Month	T min	T max	Rainfall
	(°C)		(mm)
March	6.8	15.1	55.2
April	8.0	17.7	52.4
May	12.1	21.9	23.0
June	16.3	27.6	24.4
July	20.3	30.8	63.0
August	18.4	28.7	203.8

On April 15 and June 16, 2015 fertilization with 100 kg ha^−1^ of N was carried out with a centrifugal spreader. Ammonium sulphate (21-0-0) was used as the N source. During turfgrasses growing period (May to August) water was applied every other day to compensate for 100% evapotranspiration. In the spring, turfgrasses were left unmown and from June 2015, a cutting height of 12 cm was maintained with a rotary mower and clippings were removed. Three replications (block) with 3×3 m plots each, were arranged in the canopy of each species. On August 31, September 1 and 2, 2015, spectroradiometric measurements and phytometric determinations were carried out on the two species, analyzing one replication (block) per day. Measurements were taken between 11.30 am and 1.30 pm (local time) each day, in the complete absence of cloud cover. The average solar radiation intensity between 11.30 am and 1.30 pm was measured using a Quantum meter MQ-200 (Apogee Instruments, Utah, USA) and the photosynthetic photon flux density was 1710, 1825 and 1802 μmol m^-2^s^-1^ for August 31, and September 1 and 2, respectively.

### Spectroradiometric measurements

Spectral measurements were taken with a portable spectroradiometer LI-COR1800 (LI-COR Inc., Lincoln, NE, USA), with a scan range between 300 and 1100 nm, with selectable scan intervals of 1, 2, 5 or 10 nm. In this research spectroradiometric readings were carried out in the 390–1100 nm region at 5 nm intervals.

**Spectral Transmittance (T)** was measured within the canopy fitting the spectroradiometer with a fiber-optic cable (1.5 m) and a cosine-corrected sensor with 7 mm diameter (LI-COR Inc., Lincoln, NE, USA) suitable for entering the canopy without disturbing the leaf architecture. The cosine receptor is a translucent collector which samples radiant flux according to the cosine of the incident angle, and will accept radiation from all angles of a hemisphere. The sensor was fixed to the tip of the optical fiber cable which transmitted the signal to the spectroradiometer. Transmittance was determined locating the sensor inside the canopy at different levels. In order to carry out the measurements with the sensor inside the canopy at the required heights, a purpose-designed apparatus was setup, consisting in a metal shaft on which the sensor was fixed. Spectroradiometric measurements were taken at eight levels in the canopy (levels from the ground: A (0 cm); B (1.5 cm); C (3 cm); D (4.5 cm); E (6 cm); F (7.5 cm); G (9 cm), and H (10.5 cm). A measurement of incoming radiation was acquired with the sensor above the canopy. Transmittance of each level is reported as percentage of incoming radiation. Transmittance in the PAR region has been calculated as average transmittance of single acquired bands between 400 and 700 nm.

**Spectral Reflectance (R)** was measured fitting the spectroradiometer with the fiber-optic cable and a LI-COR 1800–06 telescope (LI-COR Inc., Lincoln, NE, USA) suitable to acquire reflectance data from above the turf canopy. The telescope was mounted on a purpose-built trolley 130 cm above the ground with a vision angle set at 15°. The surface area monitored at ground level corresponded to approximately 2000 cm^2^ (diameter = 50 cm). The radiation reflected by a white panel made from barium sulphate was measured as a reference in order to detect any possible variation in irradiance. Data are reported as percentage of radiation reflected by the reference surface.

**Spectral Absorbance (A)** was calculated as: Absorbance = 1- (Reflectance + Transmittance).

### Phytometric determinations

#### Whole canopy measurements

In order to accurately sample the vegetation, a 400 cm^2^ surface of the sward was collected for each of the three replications and the following determinations were carried out:

- Shoot density: (n° cm^-2^) direct counting with data reported as shoot cm^-2^;
- Specific leaf dry weight (SLWd) (g m^-2^): mass of dry matter per unit leaf area [25]. Leaves were placed on paper, scanned and measured with the ImageJ software (imagej.net; National Institutes of Health, Bethesda, Maryland, USA).

#### Measurements by canopy layers

After spectroradiometric measurements, eight layers of phytomass located between the ground level and the top of the canopy were sampled on a surface of 800 cm^2^ (layers: 1^st^ (0–1.5 cm); 2^nd^ (1.5–3 cm); 3^rd^ (3–4.5 cm); 4^th^ (4.5–6 cm); 5^th^ (6–7.5 cm); 6^th^ (7.5–9 cm); 7^th^ (9–10.5 cm) 8^th^ (10.5–12 cm). The following phytometric determinations were performed using the stratifying clipping method suggested by [40;41].

- Leaf blade dry weight (g m^-2^): in each layer blades and blade portions were collected and then dried in a forced-air oven and weighed to determine dry biomass.
- Shoot dry weight (g m^-2^): in each layer shoot portions were collected and then dried in a forced-air oven and weighed to determine dry biomass.
- Total dry weight (g m^-2^): sum of leaf blade and shoot dry weight of each layer.
- Δ Leaf Area Index (ΔLAI): was calculated as the leaf area of each layer/ground area (m^2^ m^-2^) [29]. The leaf area index was calculated from the relationship between the leaf blade dry weight and the specific leaf dry weight (SLWd).
- Total LAI: was derived from the sum of the ΔLAI of each layer.

In addition, close to the sampling areas, on each measurement day, five vertical sections of the canopy of the two species were photographed. On the printed photos, the angles with respect to the vertical axis of 100 leaf blades were measured with a clinometer [26; 19].

### Statistical analysis

Phytometric parameters were subject to analysis of variance (ANOVA) using CoStat software (CoHort, Monterey, CA, USA).

Shoot density, specific leaf dry weight (SLWd) [25], leaf blade dry weight, shoot dry weight, total dry weight and LAI, were treated according to a randomized block design. Layer data were processed according to a factorial design assuming the species as the main factor and the layers as the secondary factor.

The relationship among cumulative LAI and the transmittance in PAR was studied by linear regression analysis using CoStat software and the determination coefficient (R^2^) was calculated.

## Results and discussion

### Phytometric parameters

#### Canopy measurements

Shoot density (n° shoots cm^-2^) and SLWd (g m^-2^) were higher in Zm (6.93 and 60.4 respectively) compared to Cdxt (2.61 and 36.7 respectively) (Table 2).

**Table 2:** Shoot density and specific leaf dry weight of *Zoysia matrella* and *Cynodon dactylon x transvaalensis*. Species mean effect.

	Shoot density^a^ (n° cm^-2^)	Specific leaf dry weight (SLWd)^a^ (g m^-2^)
Zm	6.93	60.4
Cdxt	2.61	36.7
	**	**

** = P ≤ 0.01

ns = not statistically significant

^a^ = values averaged across August 31 and September 1 and 2, 2015 sampling dates

Cdxt and Zm had a very different leaf arrangement, in particular Zm had a mean leaf angle of 22.4° (±3.4), while Cdxt presented a mean leaf angle of 62.1° (± 9.6). Thus, the canopy of Zm proved to be tendentially erectophile, while Cdxt basically presented a planophile canopy.

#### Canopy layer measurements

Analyzing the different layers of each species, the interactions between species and layers were found to be statistically significant.

A total leaf blade dry weight of 546.3 g m^-2^ (Table 3B) was recorded for Zm with higher values in the upper layers compared to those close to the ground. Conversely a lower total leaf blade dry weight (207.4 g m^-2^) (Table 3B) was recorded for Cdxt with a total absence of leaves in the first 3 layers (0–4.5 cm) (Table 3A). Consequently, the LAI value was lower in Cdxt (15.6) than in Zm (9.0) (Table 3B). With respect to the shoot dry weight, Cdxt showed higher values in all the layers with total values of 567.9 g m^-2^ for Cdxt compared to 178.6 g m^-2^ for Zm (Table 3B). In terms of total dry weight (leaf blades + shoots), the two species did not differ significantly, whereas in some layers, the two species differed significantly with respect to the total dry weight.

**Table 3:** a) Leaf blade dry weight, shoot dry weight, total dry weight (leaf blades+shoots) and ΔLAI of layers. Interaction between species and layers. b) Total leaf blade dry weight, total shoot dry weight, total dry weight (leaf blades+shoots) and total LAI (all layers). Species mean effect.

**a)**								
**Layer**	**Leaf blade** **dry weight**		**Shoot** **dry weight**		**Total dry weight** **(leaf blades+shoots)**		**ΔLAI**	
**(cm)**	**(g m**^**-2**^**)**		**(g m**^**-2**^**)**		**(g m**^**-2**^**)**			
	*Zm*	*Cdxt*	*Zm*	*Cdxt*	*Zm*	*Cdxt*	*Zm*	*Cdxt*
1^st^ (0–1.5)^a^	4.8	0	32.2	60.7	37	60.7	0.08	0
2^nd^ (1.5–3)	13.9	0	29.4	114.9	43.3	114.9	0.23	0
3^rd^ (3–4.5)	36.7	0	22.6	113.1	59.3	113.1	0.61	0
4^th^ (4.5–6)	51.0	12.6	27.8	92.3	78.8	104.9	0.84	0.34
5^th^ (6–7.5)	94.4	27.5	31.5	85.4	125.9	112.9	1.56	0.75
6^th^ (7.5–9)	131.0	49.1	25.3	61.7	156.3	110.8	2.17	1.34
7^th^ (9–10.5)	109.1	48.1	7.3	26.6	116.4	74.7	1.80	1.31
8^th^(10.5–12)	105.6	70.0	2.5	13.2	108.1	83.2	1.75	1.90
**LSD .05**	19.6		17.3		25.0		0.31	
**b)**								
	**Leaf blade** **dry weight** **(g m**^**-2**^**)**		**Shoot** **dry weight** **(g m**^**-2**^**)**		**Total dry weight** **(leaf blades+shoots)** **(g m**^**-2**^**)**		**LAI**	
Total (all layers)	546.5	207.3	178.6	567.9	725.1	775.2	9.0	5.6
	**		**		ns		**	

LAI, Leaf Area Index; *Zm*, *Zoysia matrella; Cdxt Cynodon dactylon x transvaalensis*.

**pairs of values differ significantly for P≤ 0.01

ns = not statistically significant.

^a^ = (1^st^ 0–1.5 cm) layer at ground level; (8^th^ 10.5–12 cm) layer at canopy top.

### PAR transmittance and relation to downward cumulative LAI

**Fig 1:**
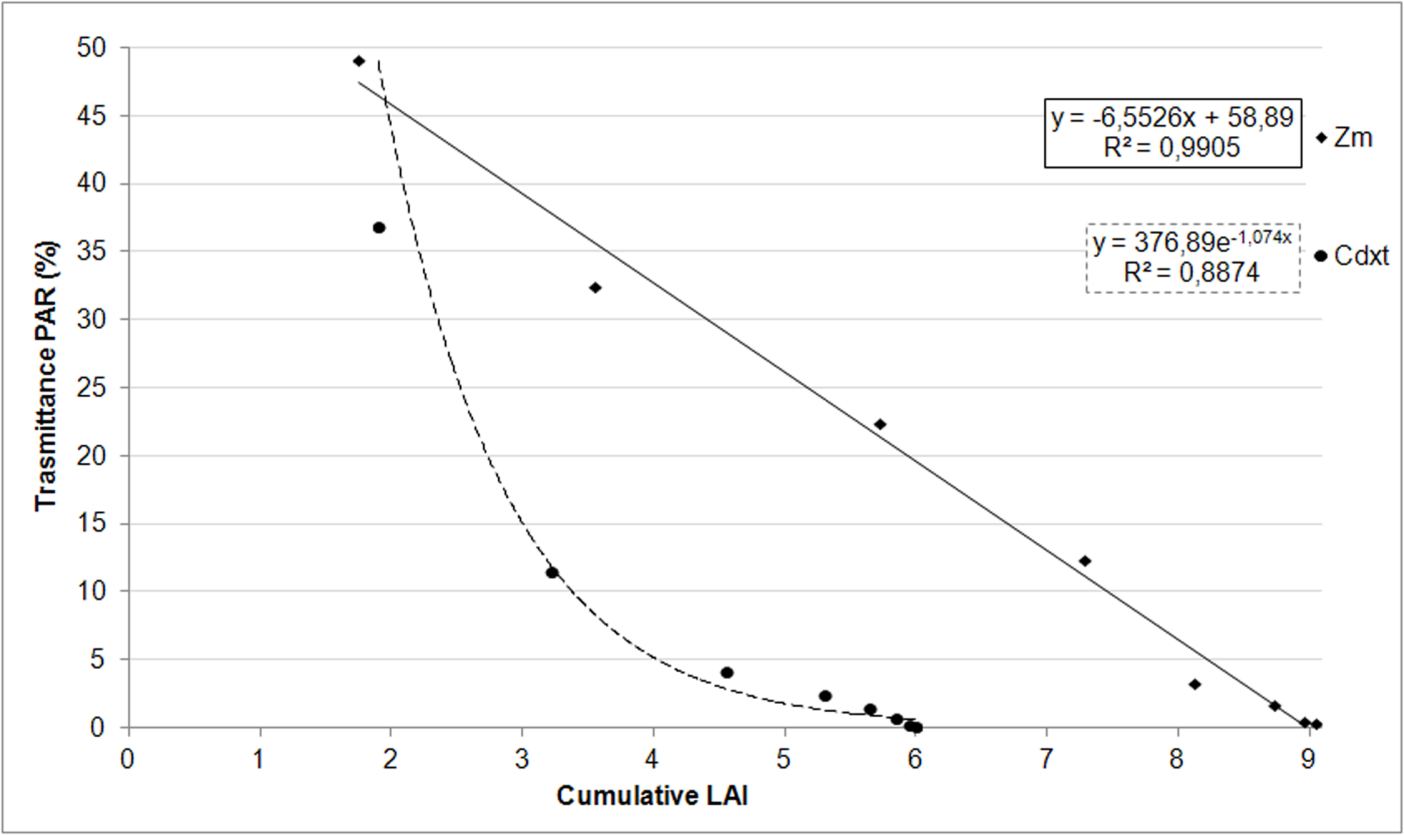
Effect of downward cumulative LAI on transmittance of PAR within the canopy of *Zoysia matrella* and *Cynodon dactylon x transvaalensis*: Recorded data and regression functions.

Regression equations between transmittance in PAR on the downward cumulative LAI for Zm and Cdxt are reported in Fig 1.

#### Zoysia matrella (Zm)

In Zm, the determination coefficient was R^2^ = 0.99. In the PAR region, the transmittance decreased linearly with increasing downward cumulative LAI, with values ranging from 49% to 0.4%, corresponding to cumulative LAI values ranging from 1.8 to 9.0 (Fig 1). Thus, Zm had a linear light extinction within the canopy.

#### Cynodon dactylon x transvaalensis (Cdxt)

For Cdxt, the exponential function proved to be the most suitable mathematical representation of the correlation between transmittance and cumulative LAI (R^2^ = 0.89). Thus, this species presented an exponential extinction of solar radiation.

In the PAR region, the transmittance values ranged from 37% to 0.2%, corresponding to cumulative LAI values ranging from 1.9 to 6.0 (Fig 1). In this species, in the lower levels (0, 1.5 and 3 cm), ΔLAI was equal to 0 due to the complete absence of green leaves (Fig 1).

#### *Zm* vs *Cdxt*

A comparison of the two species, reveals that the Zm linear light extinction within the canopy is associated with its vertical habitus of leaves, while the Cdxt exponential extinction of solar radiation is associated with the more horizontal leaf blades of this species.

As expected, the better penetration of light into Zm is also associated with the presence of green leaves in the whole profile of the canopy. On the contrary, in Cdxt light is markedly attenuated by the upper layers and a complete lack of green leaves is observed close to ground surface.

### Spectral transmittance and layers

#### Zoysia matrella (Zm)

**Fig 2:**
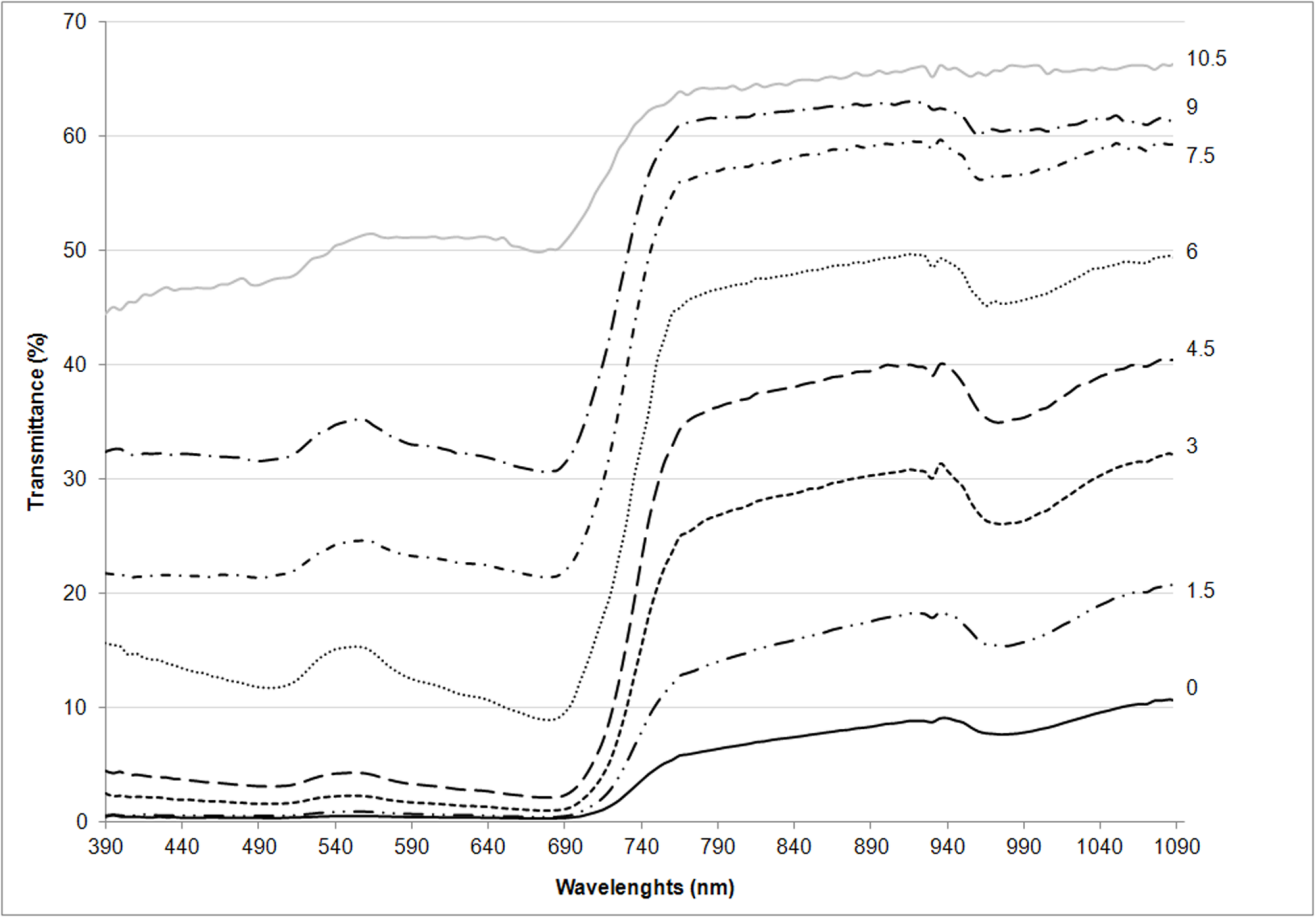
Spectral transmittance at 8 different levels within the canopy of *Zoysia matrella*: At ground level (0 cm) and at 1.5, 3.0, 4.5, 6.0, 7.5, 9.0, 10.5 cm heights (values averaged across measurements taken on August 31, September 1, September 2, 2015).

Spectral transmittance curves at eight measurement levels, in PAR and NIR regions in Zm are reported in Fig 2. As expected, moving from the upper layers to the ground level, the transmittance values decrease because the radiation is progressively absorbed and reflected.

In the PAR region of transmittance curves, values are higher between 500 and 600 nm, with a peak at 555 nm in all the levels. In the NIR region, the transmittance curve shows a depression between 940 and 1040 nm with a minimum value at about 990 nm. At the 10.5 cm level, the transmittance curve has two characteristics: an increase at the 555 nm peak; a depression between 940 and 1040 nm. These two observations are attenuated with respect to the other levels as the sensor is partially affected by the direct incoming radiation (sunflecks). Zm presents a higher percentage of transmittance at the 10.5 cm level in all the spectra, with NIR transmittance values that range between 60% and 70%.

#### Cynodon dactylon x transvaalensis (Cdxt)

**Fig 3:**
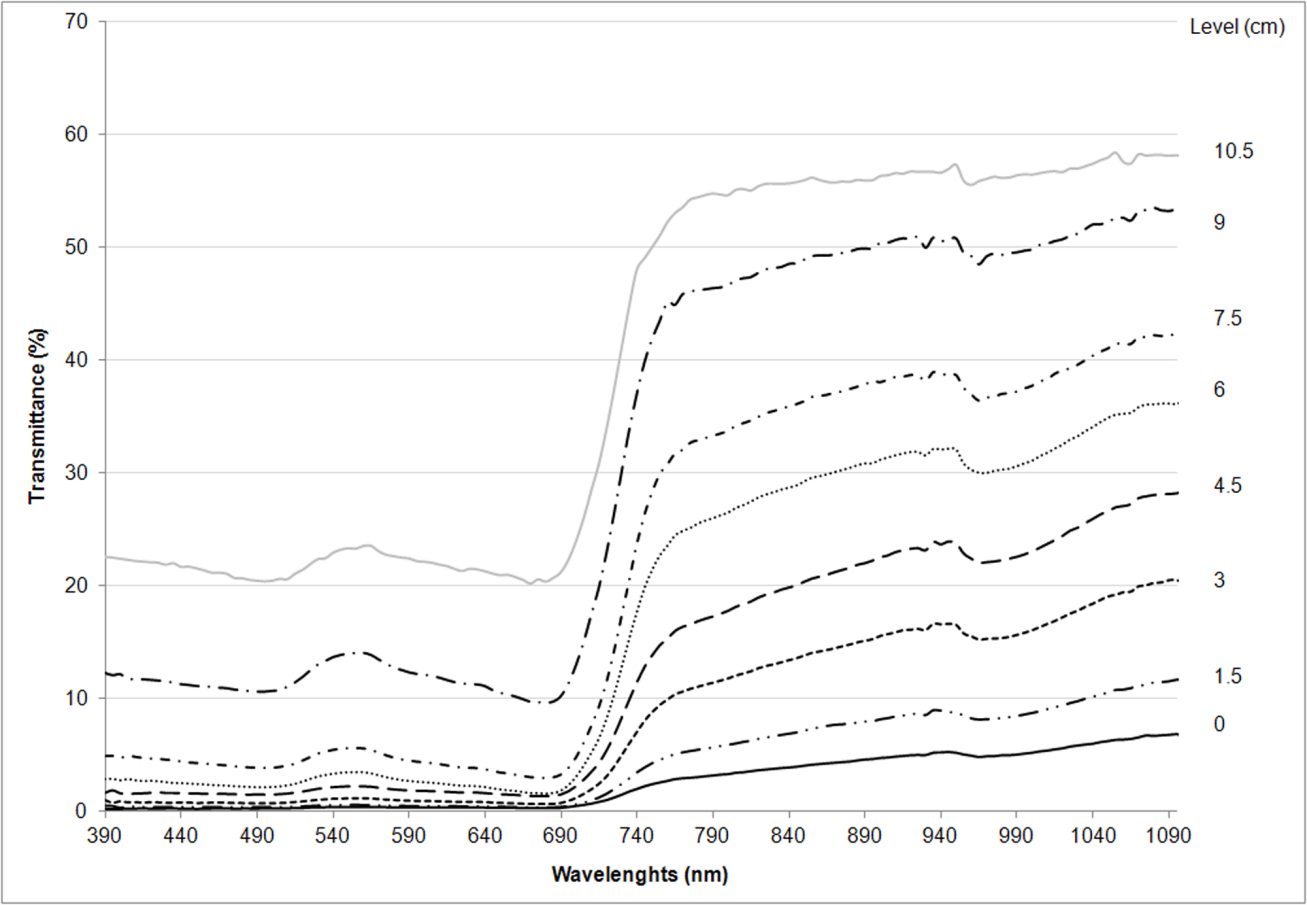
Spectral transmittance at 8 different levels within the canopy of *Cynodon dactylon x transvaalensis*: At ground level (0 cm) and at 1.5, 3.0, 4.5, 6.0, 7.5, 9.0, 10.5 cm heights (values averaged across measurements taken on August 31, September 1, September 2, 2015).

The transmittance trend in *Cynodon dactylon x transvaalensis* of all the levels where spectral measurements were taken is reported in Fig 3. In the PAR region, particularly at the chlorophyll absorption (680 nm) peak, the closer to the ground, the more values approach zero. This is because in this species, the leaves are concentrated in the upper layers, with an absence of leaves in the deeper layers, closer to the ground.

In the NIR region, the transmittance curves show a depression between 940 and 1040 nm with a minimum value at about 990 nm. Cdxt presents a higher percentage of transmittance at the 10.5 cm level in all the spectrum, with a NIR transmittance value that ranges between 50% and 60%.

### Spectral reflectance

**Fig 4:**
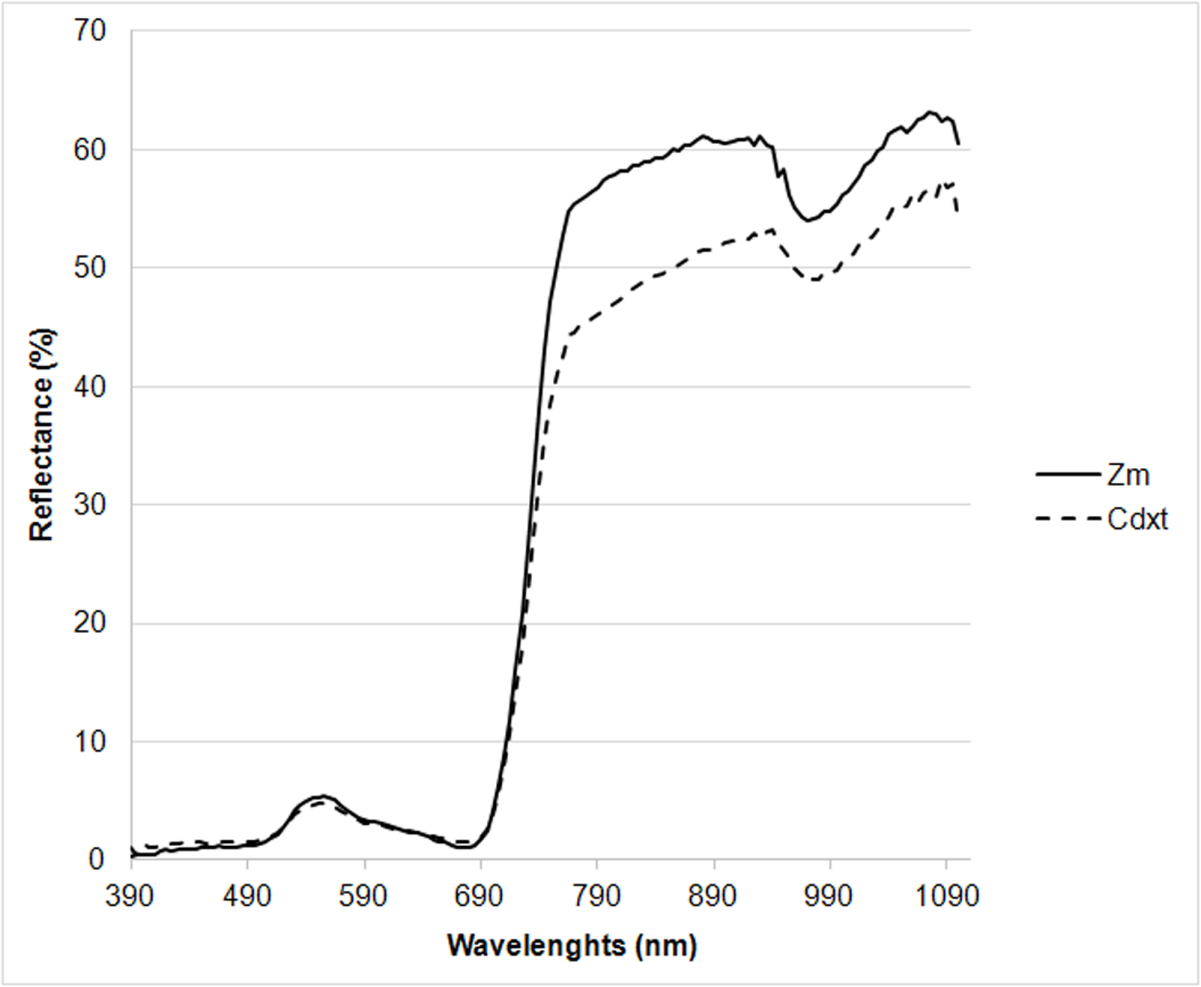
Spectral reflectance curves of *Zoysia matrella* and *Cynodon dactylon x transvaalensis* (means of measurements on August 31 and September 1 and 2, 2015).

The reflectance trend shares similar characteristics with transmittance, as incident radiation interacts deeply with the reflecting crop and is partly absorbed, transmitted and reflected only after a multitude of reflectance, refraction and diffusion phenomena, both within single leaves and the whole canopy [42]. The reflectance spectrum obtained in Fig 4 highlights the minimal differences between the two Zm and Cdxt species in the PAR region. Cdxt presents higher reflectance values in this region although at the 555 nm peak, Zm shows higher spectral reflectance values (Zm 5.5% vs Cdxt 4.8%). Furthermore, in the NIR region, the gap between *Cdxt* and *Zm* is accentuated. *Zm* presents approximately 10% higher reflectance values than *Cdxt* (Fig 4).

### Spectral absorbance

**Fig 5:**
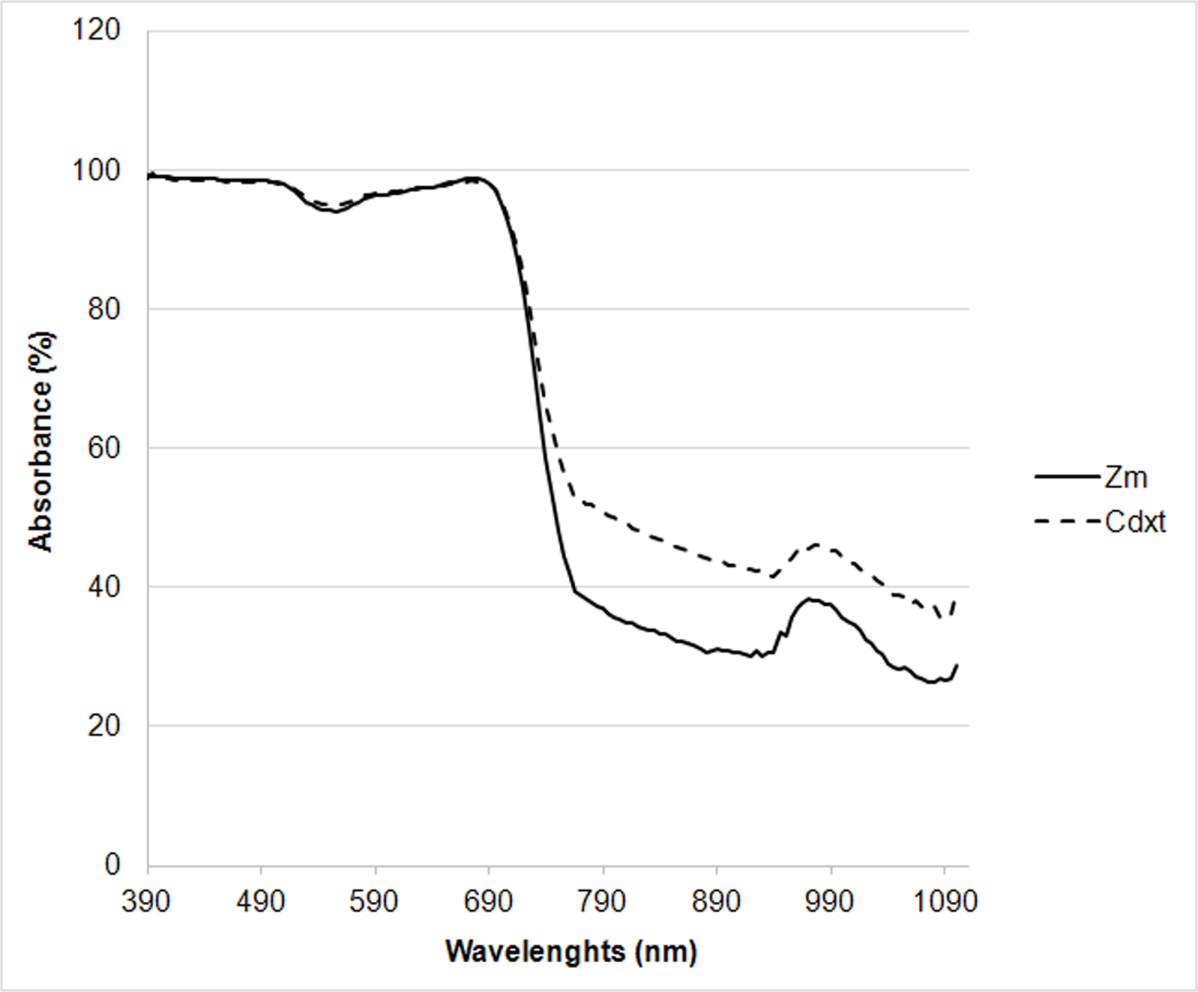
Spectral absorbance curves of *Zoysia matrella* and *Cynodon dactylon x transvaalensis* (means of measurements on August 31 and September 1 and 2, 2015).

Wavelengths close to blue (450 nm) and close to red (680 nm) were almost completely absorbed by the canopy (Fig 5). In the PAR region, absorbance values of the two species are very similar, although at the peak of 555 nm, the Cdxt value is slightly higher than Zm (Cdxt 94.9% vs Zm 94.0%).

In NIR the amount of absorbed radiation in Cdxt is clearly higher with respect to Zm (Fig 5).

In accordance with [27] and [43], in our study, due to the more erectophile habitus, Zm turfgrass showed a more efficient penetration of NIR light through the canopy layers than the planophile Cdxt species. However, the expected “light entrapment” caused by the more efficient light penetration in Zm, was not observed in NIR, and a higher reflectance and lower absorbance compared to Cdxt were instead recorded. The association reported by [28] and [43] between erectophile habitus and the efficient use of light was also not confirmed in this research in the PAR region. Different canopy architectures, as described by the phytometric data, and distinct PAR light extinction models were in fact associated with negligible differences in absorbance in the PAR region of the spectra.

## Conclusions

In this preliminary study carried out on turfgrasses mowed at 12 cm (a real situation in horse race tracks and the rough of golf courses), the species examined displayed substantial differences both in terms of phytometric characteristics and radiometric properties. The canopy of the two species presented very different architectures with a different distribution of biomass in the layers, and different angles of insertion of the leaves. The association described in the literature between canopy architecture traits and canopy radiometric properties was only confirmed for NIR transmittance. This thus indicates that variables governing light interaction with turfgrasses might be different from those studied in field crops.
